# Psychotherapy with Music Intervention Improves Anxiety, Depression and the Redox Status in Breast Cancer Patients Undergoing Radiotherapy: A Randomized Controlled Clinical Trial

**DOI:** 10.3390/cancers13081752

**Published:** 2021-04-07

**Authors:** Patrizia Zeppegno, Marco Krengli, Daniela Ferrante, Marco Bagnati, Vincenzo Burgio, Serena Farruggio, Roberta Rolla, Carla Gramaglia, Elena Grossini

**Affiliations:** 1 Psychiatry Institute, Department of Translational Medicine, University of “Piemonte Orientale” and University Hospital “Maggiore della Carità”, Via Solaroli 17, 28100 Novara, Italy; patrizia.zeppegno@med.uniupo.it (P.Z.); carla.gramaglia@med.uniupo.it (C.G.); 2 Radiation Oncology Division, Department of Translational Medicine, University of “Piemonte Orientale” and University Hospital “Maggiore della Carità”, Via Solaroli 17, 28100 Novara, Italy; vincenzo.burgio_1987@libero.it; 3 Unit of Medical Statistics, Department of Translational Medicine, University of “Piemonte Orientale” and Cancer Epidemiology, CPO Piemonte, Via Solaroli 17, 28100 Novara, Italy; daniela.ferrante@med.uniupo.it; 4 Department of Health Sciences, Clinical Biochemistry Laboratory, University of “Piemonte Orientale” and University Hospital “Maggiore della Carità”, Via Solaroli 17, 28100 Novara, Italy; marco.bagnati@maggioreosp.novara.it (M.B.); roberta.rolla@med.uniupo.it (R.R.); 5 Physiology Laboratory, Department of Translational Medicine, University of “Piemonte Orientale”, Via Solaroli 17, 28100 Novara, Italy; serefar@live.it (S.F.); elena.grossini@med.uniupo.it (E.G.)

**Keywords:** breast cancer, depression, anxiety, resilience, psychotherapy, music intervention, oxidants, antioxidants

## Abstract

**Simple Summary:**

Changes in the redox status and inflammation represent shared features among breast cancer, radiotherapy (RT)-related side effects, and mood disorders. Markers of peroxidation and inflammation are increased in patients with anxiety and depression, and their blockage could modulate these symptoms in cancer patients. The current literature about the role of psychotherapy with music intervention (PMI) in modulating anxiety, depression, and redox/inflammation status in breast cancer patients undergoing RT is still scant. To our knowledge, this is the first randomized trial showing PMI beneficial effects not only on anxiety and depression, but also on redox status. The results obtained highlight the potential of integrative therapies, specifically of PMI, as a valuable tool for the management of mood disorders in breast cancer patients undergoing RT. Findings concerning the redox status are promising and warrant further investigation.

**Abstract:**

The aim of this study was to assess the effects of psychotherapy with music intervention (PMI) on anxiety, depression, redox status, and inflammation in breast cancer patients undergoing radiotherapy (RT). This monocentric randomized clinical trial recruited 60 patients who had a breast cancer operation and were undergoing postoperative RT. Eligible patients were randomized (1:1) in two groups: the control group (CG) received treatment as usual (*n* = 30), i.e., RT alone; the intervention group (PMI) received RT and psychotherapy with music intervention (*n* = 30), which was delivered in a group setting. Five patients were excluded after randomization. Assessments were performed at baseline (T0), at the end of RT (T1), and three months after the end of RT (T2). The main objectives of the study were the assessment of anxiety/depression, plasma glutathione (GSH), and thiobarbituric acid reactive substances (TBARS) in the two arms of the study. Our findings revealed a positive effect of PMI on anxiety, depression, resilience, and quality of life. Furthermore, a positive effect of PMI on redox status was found for the first time. Thus, in the PMI group, we found a significant increase of GSH (mean change 2.2 95%, CI 0.7 to 3.7) and a significant reduction of TBARS (mean change −1.1 95%, CI −1.8 to −0.3) at T2 vs. T0.

## 1. Introduction

Breast cancer is the most common malignancy in the female population and the second most common cancer across the globe. In 2018, it was responsible for about two million cancers, accounting for the fifth leading cause of cancer deaths worldwide [1]. Adjuvant and neoadjuvant systemic treatments have significantly improved clinical outcomes [2]. The CONCORD study reported that the five-year net survival for breast cancer has steadily increased to almost 80% in many countries [3]. Recent data indicate that radiotherapy (RT) provides a significant survival advantage to patients after breast conserving surgery, despite the related side effects and toxicity [4,5]. Among various mechanisms of radiation-induced toxicity, the production of oxygen reactive species (ROS) and inflammation could account for the indirect ones [6,7,8]. In addition, increased cytokines release and oxidative stress could contribute to breast cancer recurrence and represent a key factor also for a broader cluster of symptoms, including depression as well as pain, fatigue, and sleep disturbances [9,10].

Overall, anxiety and depression may affect up to 10% and 20% of cancer patients at any point of their clinical history, leading to a worse prognosis and an increased risk for non-adherence to medications [11,12]. Several reviews reported a large spectrum of results about the impact of integrative therapies (art therapy, creative psychological interventions, music therapy interventions, etc.) in cancer patients [13,14,15]. Regarding music therapy, it should be underscored that under this label there is a wide variety of approaches. Music therapy in its widest meaning refers to the use of music, in all its forms, in the context of the therapist–patient relationship. Music therapy can be either receptive or active. In receptive music therapy, patients just listen to music, but are actively involved in perception, imagination, and elaboration under the therapist’s guidance. In active music therapy, patients directly produce sounds, singing or using instruments. Music medicine is a phrase used for an intervention (usually delivered by healthcare workers) where patients listen to recorded music. Furthermore, an even wider variety of music-based interventions exist, including all the other uses of music activities to promote health or recreational goals. Music-based interventions are usually performed by health care professionals or musicians. Preliminary evidence has supported the effectiveness of music therapy and music-based interventions (which are not always easy to disentangle in the available studies about this topic) on anxiety in breast cancer patients [16]. One study focused on the impact of music on anxiety in the context of bioptic procedures [17]. A study involving 70 women with advanced metastatic breast cancer randomized to receive three individual sessions conducted by a music therapist found significant immediate effects in terms of relaxation, comfort, happiness, and heart rate, but failed to identify significant differences between conditions over time [18]. A systematic review and meta-analysis included 13 trials about arts therapies (music therapy interventions, various types of art therapy, and dance/movement) retrieved from the Cochrane Central Register of Controlled Trials, PubMed, and Google, for a total of 606 patients, [13]. Notwithstanding the methodological quality, which ranged from poor to high, results suggested that arts therapies seemed to positively affect patients′ anxiety, but not depression or quality of life. On the other hand, different findings emerged from a more recent review, which investigated the effects on quality of life of art therapy, including music-based interventions in women with breast and gynecological cancers [19]. Findings emerging from the 19 studies eventually included in the review suggested that, overall, art therapy was effective in improving patients’ quality of life, anxiety, depression, pain, and fatigue, advocating for further studies to support these results. Overall, the available evidence suggests that music-based interventions could reduce anxiety and depression in breast cancer patients [20]. The biological mechanisms through which music-based interventions could exert beneficial effects are still debated, despite evidence about shared pathways (altered redox status and inflammation) linking RT side effects and depression.

For this reason, we aimed to analyze the effects of psychotherapy with music intervention (PMI) in a population of breast cancer patients undergoing RT, focusing on anxiety, depression, plasma oxidants and antioxidants, such as glutathione (GSH), and thiobarbituric acid reactive substances (TBARS). Moreover, we examined the changes in resilience/quality of life and plasma markers of inflammation.

## 2. Materials and Methods

### 2.1. Study Design and Participants

In this monocentric open-label parallel prospective randomized controlled trial, patients’ recruitment took place from March 2018 to June 2019 at the time of the first consultation in the Radiation Oncology Division of the “Maggiore della Carità” University Hospital in Novara, Italy. Inclusion criteria were age > 18 years, histological diagnosis of breast cancer, stage pTis, pT1–2, pN0–1 M0 (TNM classification, 2018), ECOG 0–1, lumpectomy or quadrantectomy, negative surgical margins, adjuvant radiotherapy indication, and willingness to sign informed consent. Patients were excluded in the presence of concomitant chemotherapy or targeted therapy indication, previous diagnosis of major depressive disorder, alcohol or substance abuse comorbidity, treatment with antidepressants and/or mood stabilizers, cognitive impairment or dementia, autoimmune or inflammatory diseases, diabetes mellitus, and pregnancy.

Eligible patients signed a written informed consent. Participants were informed that they could withdraw at any time during the study. Patients were treated according to the Good Clinical Practice standards [21]. This study was approved by the Ethical Committee of the “Maggiore della Carità” University Hospital in Novara (Protocol 1130/CE; Study n. CE 193/17).

After recruitment, patients were randomized (1:1) in two arms: the control group (CG) received treatment as usual (*n* = 30), i.e., RT alone; the intervention group (PMI) received RT and psychotherapy with music intervention (*n* = 30) in a group setting. Simple randomization with permuted blocks size of six was used to allocate patients to the two arms using SAS software 9.1 (SAS institute Inc., Cary, NC, USA, 2009). Allocation was done using a computer random number generator by the study statistician. The assignment of participants to a random sequence was undertaken by the radiation oncologist. Patients and clinicians, except the study statistician who was masked, were not masked to treatment assignment due to the nature of the intervention. The study is registered at ClinicalTrials.gov, NCT04446624.

### 2.2. Procedures

CG and PMI groups received the ordinary radiation treatment planned for breast cancer patients, i.e., irradiation of residual breast following standard guidelines. After Computed Tomography (CT)-simulation, patients were scheduled to receive 20–25 RT sessions to a total dose of 40.5–54.0 Gray (Gy) with dose per fraction ranging from 2.0 to 2.7 Gy to the whole breast +/− boost dose on tumor bed. Every five treatments (once a week) and on the last day of treatment, all patients had a clinical evaluation by a radiation oncologist to assess patient compliance and the onset of early side effects, usually skin erythema scored by using the Radiation Therapy Oncology Group (RTOG) scale [22]. The PMI group was offered, besides treatment as usual, six weekly sessions of brief group psychodynamic psychotherapy with music intervention during the RT course. The group included a minimum of six and a maximum of eight patients. The group format was also chosen because of its added value as far as social and peer support are concerned [23]. All participants in the PMI group received the same intervention, the same number of sessions, and the same recorded music. With more detail, each participant in the PMI arm received six sessions over a six-week period; each session lasted one hour. The PMI intervention was delivered at the Psychiatry Institute of the “Maggiore della Carità” University Hospital, Novara, Italy. A large room was used to host the group, where participants and therapists could sit in a circle. The environment was quiet and granted a proper privacy level. PMI was conducted by a psychiatrist with more than ten years of experience in psychodynamic group psychotherapy with Jungian orientation. Two residents in training participated in the PMI sessions, with a supportive role. Music was pre-selected by the group leader; in every session, an Italian song was proposed for listening to participants (a list of the songs used will be available upon request). Songs were chosen from the Italian pop repertoire and from that of Italian classical songwriters. Italian songs were chosen to allow a therapeutic work on two levels: a deeper one, elicited by music and by its potential to evoke emotions and memories, and a more “rational” one, elicited by lyrics (Italian language enabled all participants to clearly understand the meaning of the song), allowing a reflection of the themes emerging from the songs. The choice of songs that were likely to be known (as they are very famous in Italy) to most participants in the group was made in order to enhance the possibility of recalling memories and emotions associated to the song itself, in past or current times. Furthermore, we chose songs dealing with universal themes like love, life, courage, fear, and hope, so that also the more “rational” work on the lyrics could eventually allow talking about emotionally relevant topics for patients. Briefly, we used a combination of music listening and lyric analysis. The songs were listened to with the aid of a laptop and wireless and Bluetooth speakers; each patient received a printed version of the song lyrics. The volume of songs was controlled by the therapist and was the same for all participants, and the volume control was executed to limit decibel to 60. Consistently with the aims of the therapeutic intervention, after listening to the song, sharing of emotions, feelings, memories, and sensations elicited by music and lyrics was encouraged, and these were discussed in the group setting. Eventually, before closing the session, the song was listened to again and simultaneously sung by the whole group together, with the aim of fostering the group cohesion and offering relief.

All patients underwent a psychiatric assessment and blood sampling for dosing redox status/inflammatory markers (as described below) at baseline, prior to the intervention (T0), at the end of RT (T1), and three months after the end of RT (T2).

Psychometric assessment was performed by the following questionnaires and scales.

State-Trait Anxiety Inventory 1 and 2 (STAI Y1, STAI Y2) [24]: a 40-item self-administered test for the assessment of state and trait anxiety. Each item is rated from 1 to 4 (1 = not at all, 4 = severe), and no specific cut-offs exist. The higher the score, the greater the anxiety.

Montgomery–Asberg Depression Rating Scale (MADRS) [25]: a 10-item clinician-rated scale for depressive symptoms detection and severity assessment. Each item is rated on a six-point scale; hence, 60 is the maximum total score indicating the maximum severity of depressive symptoms. A score < 6 points stands for no depression (normal), 7–19 for mild depression, 20–34 for moderate depression, and ≥35 for severe depression.

Beck Depression Inventory (BDI-II) [26]: a 21-item self-administered test for the evaluation of subjective depressive feelings or symptoms. It is rated on a four-point scale (from 0 to 3), with the following cutoff values: 0–9 for minimal depression, 10–18 for mild depression, 19–29 for moderate depression, and 30–63 for severe depression.

Resilience Scale for Adults (RSA) [27]: a 33-item self-administered scale evaluating intra- or inter-relational stress preventing factors (positive self-perception, positive future perception, social competence, structured style, family cohesion, and social resources). The higher the total score, the greater the subject’s resilience.

Short Form-36 (SF-36) [28]: a self-administered test assessing health-related quality of life, both from a somatic and psychological standpoint, including physical activity, role and physical health, physical pain, general health, vitality, social activities, role and emotional state, mental health, and health change. Results are expressed in age-calculated percentiles.

Blood samples were analyzed by the Physiology Laboratory, Department of Translational Medicine, University of “Piemonte Orientale” and by the Clinical Biochemistry Laboratory, “Maggiore della Carità” University Hospital, Novara, Italy. For the determination of glutathione (GSH), malondialdehyde (MDA), interleukin 6 (IL-6), tumor necrosis factor α (TNFα), and the other antioxidants and inflammation variables, 10 mL blood samples were taken from each participant by using BD Vacutainer tubes (sodium heparin as anticoagulant). GSH measurement was performed by using the Glutathione Assay Kit (Cayman Chemical, Ann Arbor, MI, USA), whereas TBARS were determined as MDA release by using the TBARS assay Kit (Cayman Chemical) [29]. The reading was performed through a spectrophotometer (VICTOR™ X Multilabel Plate Reader) at excitation/emission wavelengths of 405–414 nm for GSH and 530–540 nm for TBARS. IL-6 and TNFα measurements were performed by using the Human IL-6 and TNFα ELISA kits (Invitrogen, Carlsbad, CA, USA) through a spectrophotometer (VICTOR™ X Multilabel Plate Reader) at wavelengths of 450–620 nm. High sensitivity C-Reactive Protein (CRP) was analyzed on ADVIA^®^ 1800 Clinical Chemistry Analyzer (Siemens Healthcare Diagnostics, Milan, Italy). α and γ tocopherol, lycopene, and carotenoids levels were assessed on Agilent Eclipse XDB. Detection of lipid soluble antioxidants was performed at a specific wavelength: retinol (326 nm), α and γ-tocopherol (292 nm), and lycopene and β carotene (460 nm). Details about plasma measurements are reported in Supplementary Material-Plasma Measurements.

### 2.3. Outcomes

The primary outcome measure was anxiety based on the total score of the STAI test. Key secondary outcomes included depression, plasma levels of GSH, and TBARS. Exploratory secondary outcomes were resilience, quality of life, plasma cytokines (IL-6, TNFα), retinol, α and γ-tocopherol, lycopene, β carotene, and CRP from baseline (T0) to last follow-up (T2).

### 2.4. Statistical Analysis

The sample size calculation was based on the STAI test because of the available information in the literature about the effectiveness of music-based interventions on anxiety in breast cancer patients, as detailed in the introduction [13,16,19]. Therefore, the STAI total score was the main endpoint of our analysis. Data available from literature reported a value of 46.2 points for the experimental group (SD = 11.0) and a value of 43.4 (SD = 10.6) for the control group with the STAI test, expecting a clinically significant reduction of nine points in the experimental group and no reduction in the control group. Considering these data and an effect size equal to 0.80, it would have been necessary to recruit 24 patients per group, and, consequently, considering also possible dropouts, sample size was eventually fixed at 60 patients (alpha: 0.05; beta: 0.20) [30]. Quantitative data were presented as mean and standard deviation. Categorical variables were summarized as counts and percentages. Differences in means between the two groups were evaluated using Student’s t-test for characteristics of patients such as age and radiotherapy duration. Associations between categorical variables were tested using Pearson’s Chi-square test or Fisher’s exact test. Psychometric and laboratory data were analyzed through repeated-measures ANOVA. This method of analysis was used because of the multiple between responses taken in sequence on each experimental unit. The objective was to examine and compare response trends over time, comparing groups averaged over time and comparing measurement time within a group. The unstructured covariance matrix was used as a correlation pattern among responses to the same subject. The model used in this analysis was μ_ik_ = μ +α_i_ + г_k_ + (αг)_ik_, where μ is the overall mean, α is the “group effect”, г is the “time effect”, and (αг) the “group × time effect”.

Correlation between psychometric measures and redox status was tested using Pearson’s correlation coefficient. *p*-values were considered statistically significant when <0.05. Analysis was conducted using the software SAS (Release 9.2, by SAS Institute Inc., Cary, NC, USA) and STATA v.14 (StataCorp LP, College Station, TX, USA).

## 3. Results

**Figure 1:**
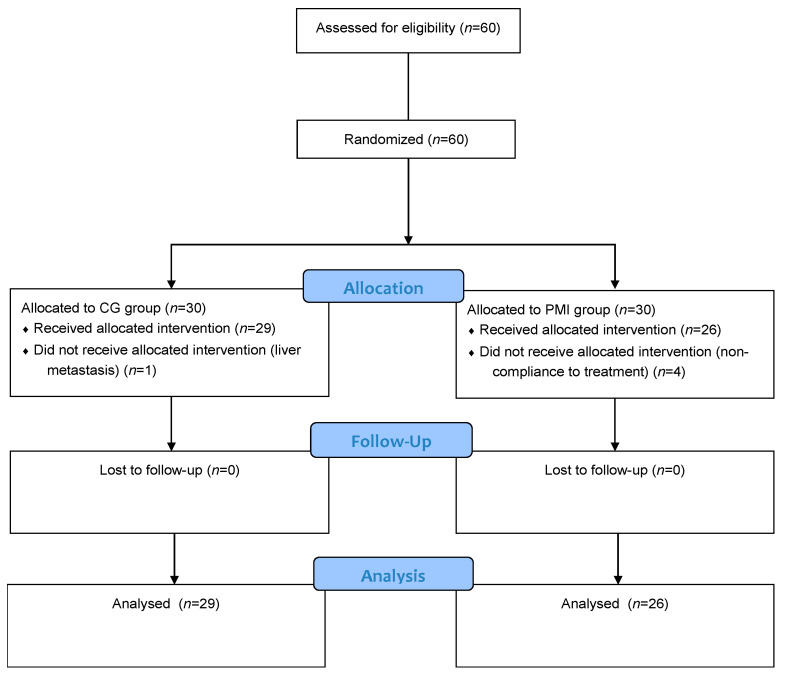
Randomized Clinical Trial profile. PMI: psychotherapy with music intervention group; CG: treatment as usual group.

Patient recruitment started in March 2018 and ended in June 2019 after enrollment completion. Sixty patients, aged 42–85 years, were enrolled in the study and randomly assigned to the CG (30 patients) and PMI (30 patients) groups. Five patients were excluded after randomization, one in the CG group and four in the PMI group, because of non-adherence to RT treatment. In particular, among the four patients belonging to the PMI group, three were excluded for poor compliance and one for onset of liver metastasis (Figure 1). Therefore, the analysis included 29 patients in the CG group and 26 in the PMI group.

Participants’ baseline characteristics are described in Table 1. No significant differences between the two groups were found in demographic data and clinical features. In addition, no significant differences in socio-educative pattern (civil status, education, and employment), history of psychotherapy, or musical practice were observed between PMI and CG groups.

Radiation-induced early side effects in terms of skin erythema grade 1–2 (RTOG scale) occurred in all patients: 21 cases (72.4%) of grade 1 and eight (27.6%) of grade 2 in the CG group, and 20 cases (76.9%) of grade 1 and six (23.1%) of grade 2 in the PMI group.

### Outcome and Estimation

Primary and secondary outcomes measured from T0 to T2 are reported in Table 2, Table 3 and Table 4.

Statistically significant differences emerged between the CG and PMI groups on several parameters. In particular, STAI Total, STAI I, and STAI II scores were lower in the PMI group than in the CG one, which showed a reduction of the primary endpoint, which was anxiety, at three months after the end of RT in the PMI patients.

Moreover, it is also notable that MADRS and BDI scores showed a significant time effect, independent from treatment and with an overlapping trend in the two groups of CG and PMI patients. In the PMI group, the scores turned from those of a mild depression at T0 into those of a normal or minimal depression at T2. Furthermore, the PMI group self-reported lower depression scores measured by BDI at T1 and T2 than the CG one. Time trends were similar for anxiety and for depression.

In the PMI group, several RSA subscale scores were higher than in the CG one, which highlighted increased resilience in the PMI group (Table 4). A significant interaction group × time emerged for all RSA subscales, except for RSA-SR (*p* = 0.059), and a significant group effect was found for RSA-FC and RSA-SR, with the PMI group scoring higher than the CG one.

A significant interaction group × time emerged for all the SF-36 subscales, except for SF-36 RP; a group effect emerged for SF-36 general health perception (GH), SF-36 social functioning (SF), and SF-36 role limitations due to emotional problems (RE). Even though in most cases (except for SF-36 RE) their baseline scores were lower than those of the CG group, patients in the PMI group eventually showed higher scores at T1 and T2 (Table 4).

**Figure 2:**
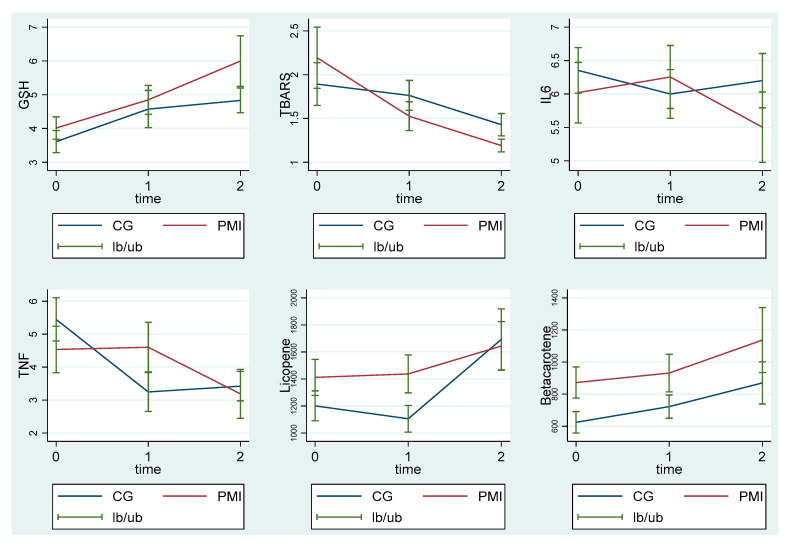
Plasma levels of glutathione, thiobarbituric acid reactive substances, interleukin 6, tumor necrosis factor α, and lycopene and β carotene in MI and CG measured at T0, T1, and T2. GSH: glutathione; TBARS: thiobarbituric acid reactive substances; IL6: interleukin 6; TNF α: tumor necrosis factor α; PMI: psychotherapy with music intervention group; CG: treatment as usual group. T0: baseline; T1: at the end of radiotherapy; T2: three months after the end of radiotherapy. The graphs show the mean and the standard error.

Similarly, changes in secondary outcomes related to inflammation are reported in Table 4 and Figure 2.

As reported in Table 4 and Figure 2, baseline values of oxidants and antioxidants or cytokines at T0 did not differ between CG and PMI groups. Therefore, the baseline values of the redox status or inflammatory parameters did not differ between patients who underwent usual treatment (CG) or psychotherapy with music therapy elements (PMI). Instead, it is worth noting that GSH and TBARS changed significantly in the PMI patients at T2 from what was found at T0 (*p* = 0.008 and *p* = 0.038, respectively), indicating that the antioxidants increased and the oxidants decreased. The improvement in the redox status was not observed in the CG group. In contrast, at T2 in the PMI group plasma, GSH levels were higher, whereas plasma TBARS levels were lower than those observed in the CG group (Table 4; Figure 2).

Regarding cytokines or CRP, no differences were observed either from T0 to T2 (time effect) or for the comparison between the CG and PMI groups. Moreover, we did not find any significant correlation between psychometric measures and redox status (Table S1). Given the type of intervention of our study, we did not expect specific harms, but rather potential benefits in the PMI group.

## 4. Discussion

Patients with breast cancer are often treated with complementary and integrative therapies as supportive care, to help them cope with anxiety, depressive symptoms, and a sense of loss of health they may experience as a consequence of diagnosis and treatment [31]. Among the various available interventions, as described in the introduction, music-based interventions could represent a valuable tool for the management of anxiety and depression symptoms in breast cancer patients [13,14,15]. Although the exact mechanisms underlying their efficacy and effectiveness are not thoroughly understood, the most commonly accepted theories revolve around neurologic, psychological, behavioral, and physiological pathways [32]. Interestingly, both alterations in redox status and inflammation could represent a pathophysiological link between cancer, depression, and RT-related side effects [3,7]; RT-associated inflammation may potentially lead to depression, which could be a further way through which radiation toxicity could manifest, especially in women with breast cancer.

Notwithstanding the limitations that will be described below, the results obtained in our study through STAI, MADRS, and BDI scores showed that the intervention offered to the PMI group was effective in reducing anxiety, self-perceived depression levels, and clinician-rated ones as well. The effect of the PMI on depression, which was not only subjective, but also objectively identified, was maintained even beyond its actual administration time, and the same was true for trait anxiety. Interestingly, the intervention produced changes to trait rather than on state anxiety, i.e., to that level of discomfort and tension that a person experiences in daily life, not depending on external factors or events.

These findings concerning anxiety and depression are particularly important, since breast cancer is now mainly a long course curable disease and depression can be burdensome for patients [31]. Although complementary and integrative therapies are often recommended for the management of breast cancer patients, evidence supporting their use in the oncology setting is still limited and sometimes inconsistent [20]. Among the various therapeutic approaches, the effectiveness of music-based interventions has been supported by some clinical trials [15,16,19,31]. Actually, an evidence-based guideline produced by the Society for Integrative Oncology (SIO) [33] on the use of integrative therapies included music therapy (in its broader meaning) among the key recommendations. Music-based approaches were considered particularly suitable for anxiety/stress reduction and depression/mood disorders, and their impact on such clinical variables has been confirmed also by a recent review [20].

Regarding resilience, in general, the results obtained with the RSA subscales (the RSA-FC subscale, which assesses family-cohesion and perception of the possibility for family members to rely on one other in difficult situations, and the RSA-SR subscale, which evaluates how a person can lean on social support given by people outside the family as friends, confidants, other supportive figures [27]) are consistent with the fact that in the PMI group, the therapeutic work on interpersonal relationships is an intrinsic component of treatment [23]. Resilience is a complex construct encompassing personal psychological and attitudinal aspects (for example, good self-perception and proactivity) and good familial and social support that enables individuals to positively cope with difficulties and stressful life events, and to be able to rebuild their own life even after critical events [27]. The significant group effect and the higher resilience scores in the PMI group suggest that the music-based intervention was able to enhance individual skills in facing and overcoming adversities, which may help patients to deal with the challenging situation they are living through.

As already underscored for self-rated depression and trait anxiety, the effect of the PMI intervention continued also during the follow-up, confirming a durable benefic effect on patients’ psychological resources. Furthermore, improving resilience and coping strategies may be determinant in reducing anxiety and improving adherence to therapies.

Finally, several significant results emerged for the SF-36 subscales, which assessed patients’ quality of life levels and global psychological condition. These results suggest that the PMI had an impact on patients’ quality of life, encompassing both physical and psychological aspects. As described above for resilience, it is likely that the group format in which the PMI was delivered proved particularly beneficial for social issues and for coping with emotional problems as well. The PMI seemed effective in improving overall QoL perception, and it played a positive role during the administration time period, as well as during the follow-up. This could provide a long-lasting benefit for patients’ oncological course, considering that overall physical and mental health status are relevant for treatment adherence and, consequently, for prognosis [34].

To our knowledge, this is the first study focusing not only on clinical and psychometric data, but also on biological ones in a sample of breast cancer patients undergoing RT and randomized into two arms, PMI and CG. In PMI patients, but not in CG ones, we observed for the first time a reduction of plasma markers of lipid peroxidation, the thiobarbituric reactive substances measured as MDA [29], and an increase of the antioxidant agent, GSH, three months after the end of RT. No significant differences were observed between the two groups of CG and PMI patients regarding cytokines release or CRP, but we cannot exclude that the lack of significant results could be due to the small sample size, as discussed in the limitations section.

We observed the modulation of the imbalance between peroxidation and its detoxification mechanism, which is typical in subjects suffering from depression or post-traumatic stress disorder. It is widely accepted that the overproduction of reactive oxygen and nitrogen species under conditions of perturbed antioxidant defenses can lead to protein and lipid peroxidation, increased blood–brain barrier permeability, and neuroinflammation. These may contribute to the changes in brain function and morphology, as observed in mental illnesses [35].

Findings about plasma GSH are of special interest, since this is one of the main systems being dysregulated in those subjects. Our results are consistent with the widely acknowledged role of oxidative stress in the onset of mood disorders, but could also shed light on the mechanisms underlying the effectiveness of PMI in breast cancer patients undergoing RT. In this context, it is noteworthy that a decrease of TBARS and an increase of GSH emerged only in the PMI group patients, who also showed an improvement in terms of anxiety, depression, and resilience.

Plasma levels of carotenoids, such as lycopene and β-carotene, increased both in PMI and CG groups from T0 to T2. No difference between the two groups was found for other antioxidants such as α- and γ-carotene and vitamin A either, or for the inflammatory marker, CRP. As described above, this lack of significant results could be ascribed to the small sample size. A larger sample and a thorough assessment of patients’ dietary habits (which was not performed in the current study) might allow the identification of further significant results, which could be particularly interesting, given that dietary components such as capsaicin, isoflavones, and carotenoids have shown inhibitory effects on cancer cells through their actions as antioxidants and anti-inflammatory agents [36,37]. In this regard, lycopene consumption would be related to decreased proliferation rate of breast cancer cells and to lesser prevalence and severity of fatigue in patients [37]. We cannot exclude that the PMI may have exerted beneficial effects on the overall health of the PMI group, including dietary habits. Nonetheless, further studies specifically focusing also on this issue are warranted.

In our population, confounding variables related to additional risk factors and clinical features were avoided, which allowed us to examine in a more controlled and direct way the effects of PMI on the study outcomes. To fulfil our purpose, validated psychometric scales and main ELISA and colorimetric assays widely used for the measurements of plasma GSH, TBARS, cytokines, and vitamins were adopted and used in an integrated way.

The current study has some limitations. First, it cannot be excluded that a larger sample size might increase the significance of some results and the strength of correlations among measured variables. Nonetheless, the sample size was properly calculated by means of a specific statistical power analysis considering reported values for the anxiety evaluation (STAI test), which was the primary outcome with available literature data. It is also notable that our sample size exceeded the one established as the lowest necessary to reach statistical significance (24 patients per group), with only five dropouts. Furthermore, since we offered patients brief group psychotherapy with music-based intervention, it is difficult to disentangle the effects of group psychotherapy and music, so it is possible that our findings depend on psychotherapy, on music, or on both. Furthermore, due to the intrinsic nature of psychodynamic psychotherapy, its exact replication is hard, and the same can be stated about the group format.

A few general comments can be made about this study and similar ones involving music-based interventions. As we have described, our intervention cannot be equated to music therapy, as it was based on a psychodynamic psychotherapy approach with music intervention. Hence, its results cannot be compared to those of music therapy, but rather to those of music-based interventions. Nonetheless, music-based interventions are highly heterogeneous, and this might limit the possibility of generalizing results. Notwithstanding these limitations, the effectiveness of this therapeutic approach has received support in the existing literature [15,16,19,21,38]. Furthermore, it should be considered that any additional interactive intervention aimed at offering patients empathic listening and attention could have a positive impact on patients’ anxiety, depression, and perceived QoL. This limits the possibility to clearly and univocally attribute the changes and effects we observed to the specific intervention we performed. While this can be a limitation from the research standpoint (albeit shared by all studies of this kind), we believe it has relevant clinical implications, as it can encourage clinicians to consider adding a supportive/integrative intervention to standard care, with the aim of improving patients’ overall psychological wellbeing. Last, further limitations include the fact that other oxidants/antioxidants or inflammatory markers (F2 isoprostanes, 8-hydroxy-2′-deoxyguanosine, IL-1, IL-4, and advanced oxidation protein products) could have been measured, to further deepen the understanding of the relationship between PMI, redox status, and mood disorders. Moreover, the limited duration of the follow-up period does not allow an assessment of a longer-term preservation of the changes in anxiety and mood symptoms and in the redox status, as well as the evolution of the disease or the appearance of RT-related late side effects.

## 5. Conclusions

This multi-disciplinary study involved clinical and physiological expertise with the aim to better understand the possible mechanism underlying PMI effectiveness in a real-world clinical setting. Overall, our findings are consistent with those of previous studies and guidelines suggesting that music therapy might be helpful in reducing anxiety and depressive symptoms [15,16,17,29,36] in breast cancer patients. The importance of implementing supportive/integrative interventions for a holistic approach to patients with breast cancer undergoing standard care is supported. As this study was a preliminary attempt to match clinical, psychometric, and biological data in this field, it suggests further avenues for future research.

## Figures and Tables

**Table 1:** Characteristics of the participants.

Sample Feature	CG Group (*n* = 29)	PMI Group (*n* = 26)	*p*-Value
**Civil Status**			
Married	14 (48.2)	19 (73.0)	
Unmarried	15 (51.8)	7 (27.0)	0.06
**Education**			
Lower-middle	18 (62.0)	15 (57.7)	
Upper	11 (38.0)	11 (42.3)	0.74
**Employment**			
Non employed/retired	25 (86.2)	23 (88.4)	
Employed	4 (13.8)	3 (11.6)	0.80
**Previous Psychotherapy/Musical** **Practice**	0	0	-
**Age at Enrollment**	66.6 (10.9)	63.0 (10.7)	0.22
**Smoking ^a^**			
Yes	4 (13.8)	3 (11.5)	1.00
No	25 (86.2)	23 (88.5)	
**Stage ^a^**			
Tis	4 (13.8)	6 (23.1)	0.67
T1	22 (75.9)	18 (69.2)	
T2	3 (10.3)	2 (7.7)	
**Histologic Type ^a^**			
Ductal carcinoma	25 (86.2)	22 (84.6)	1.00
Lobular carcinoma	4 (13.8)	4 (15.4)	
**Radiotherapy Duration ^b^ (days)**	26.0 (5.5)	28.4 (4.9)	0.10

CG indicates patients who received treatment as usual; PMI indicates patients who received psychotherapy with music-based intervention. ^a^: results are shown as absolute numbers; percentages in brackets. ^b^: results are shown as mean (SD).

**Table 2:** Primary outcome: anxiety (STAI TOT).

STAI TOT	CG Group Mean (SD) (*n* = 29)	PMI Group Mean (SD) (*n* = 26)	*F* (*p*-Value)
T0	85.7 (22.0)	82.8 (18.2)	Time effect: 7.73 (0.001)
T1	80.3 (20.7)	81.0 (17.6)	Group effect: 3.96 (0.052)
T2	83.5 (17.8)	62.3 (16.5)	Interaction group × time: 9.33 (0.0003)

PMI: psychotherapy with music intervention group; CG: treatment as usual group. T0: baseline; T1: at the end of radiotherapy; T2: three months after the end of radiotherapy. The *p*-values of time effect, group effect, and interaction group × time are shown.

**Table 3:** Key secondary outcomes: anxiety (STAI I and STAI II), depression (MADRS and BDI), and plasma redox status parameters (GSH and TBARS).

Outcome	CG Group Mean (SD) (*n* = 29)	PMI Group Mean (SD) (*n* = 26)	*F* (*p*-Value)
**STAI I**			
T0	43.4 (12.1)	41.8 (10.7)	Time effect: 5.07 (0.01)
T1	39.9 (11.3)	41.7 (9.2)	Group effect: 1.91 (0.173)
T2	41.9 (9.0)	33.0 (8.1)	Interaction group × time: 7.87 (0.001)
**STAI II**			
T0	42.3 (10.7)	41.0 (9.3)	Time effect: 7.46 (0.001)
T1	40.4 (11.0)	39.3 (9.7)	Group effect: 5.52 (0.023)
T2	41.6 (10.3)	29.4 (10.3)	Interaction group × time: 7.71 (0.0012)
**MADRS**			
T0	10.2 (8.2)	8.6 (5.7)	Time effect: 5.39 (0.0074)
T1	7.4 (6.5)	6.4 (3.5)	Group effect: 3.27 (0.076)
T2	8.1 (5.4)	4.5 (3.4)	Interaction group × time: 2.07 (0.136)
**BDI**			
T0	12.2 (10.7)	10.2 (6.6)	Time effect: 3.39 (0.041)
T1	9.5 (6.4)	6.6 (5.5)	Group effect: 4.93 (0.031)
T2	11.0 (5.8)	5.9 (6.9)	Interaction group × time: 1.37 (0.263)
**GSH (µM)**			
T0	3.6 (1.7)	4.0 (1.6)	Time effect: 13.77 (<0.0001)
T1	4.6 (2.7)	4.8 (2.0)	Group effect: 1.52 (0.223)
T2	4.8 (2.0)	6.0 (3.8)	Interaction group × time: 0.62 (0.542)
**TBARS (µM)**			
T0	1.9 (1.2)	2.2 (1.7)	Time effect: 5.83 (0.005)
T1	1.8 (0.9)	1.5 (0.8)	Group effect: 0.08 (0.782)
T2	1.4 (0.7)	1.2 (0.4)	Interaction group × time: 0.94 (0.395)

GSH: glutathione; TBARS: thiobarbituric acid reactive substances. Abbreviations are as in Table 2.

**Table 4:** Exploratory secondary outcomes: resilience (RSA), quality of life (SF), inflammatory markers, and biochemical variables.

Outcome	CG Group Mean (SD) (*n* = 29)	PMI Group Mean (SD) (*n* = 26)	*F* (*p*-Value)
**RSA PS**			
T0	18.3 (1.6)	17.7 (2.3)	Time effect: 0.60 (0.553)
T1	18.0 (2.3)	18.1 (1.8)	Group effect: 3.11 (0.084)
T2	16.3 (2.7)	19.0 (2.3)	Interaction group × time: 7.51 (0.001)
**RSA PF**			
T0	11.5 (2.5)	11.4 (2.6)	Time effect: 0.60 (0.551)
T1	11.2 (2.4)	11.7 (2.8)	Group effect: 3.70 (0.060)
T2	10.3 (2.0)	13.2 (3.3)	Interaction group × time: 9.08 (0.0004)
**RSA SS**			
T0	11.2 (2.8)	10.9 (2.9)	Time effect: 1.19 (0.313)
T1	10.9 (2.4)	11.3 (3.0)	Group effect: 1.99 (0.164)
T2	10.4 (2.0)	13.0 (4.2)	Interaction group × time: 4.76 (0.012)
**RSA SC**			
T0	17.0 (3.6)	15.4 (2.6)	Time effect: 0.03 (0.969)
T1	16.3 (3.8)	16 (2.5)	Group effect: 0.06 (0.806)
T2	14.9 (3.6)	17.3 (3.0)	Interaction group × time: 8.12 (0.001)
**RSA FC**			
T0	20.5 (2.0)	21.0 (2.8)	Time effect: 1.43 (0.249)
T1	19.6 (3.6)	21.5 (2.5)	Group effect: 12.01 (0.001)
T2	17.6 (4.4)	22.1 (4.1)	Interaction group × time: 6.42 (0.003)
**RSA SR**			
T0	18.2 (1.9)	19.0 (2.4)	Time effect: 0.02 (0.981)
T1	18.2 (3.7)	19.0 (1.8)	Group effect: 7.28 (0.009)
T2	17.0 (3.8)	20.0 (3.1)	Interaction group × time: 2.99 (0.059)
**RSA TOT**			
T0	112.7 (15.9)	116.5 (14.9)	Time effect: 39.55 (<0.0001)
T1	110.4 (23.2)	116.0 (17.8)	Group effect: 12.57 (0.001)
T2	86.6 (12.8)	104.6 (16.3)	Interaction group × time: 5.20 (0.009)
**SF 36 PF**			
T0	79.5 (26.1)	78.8 (30.6)	Time effect: 1.49 (0.235)
T1	75.9 (24.2)	84.8 (20.5)	Group effect: 1.30 (0.260)
T2	71.5 (25.0)	85.2 (20.7)	Interaction group × time: 8.41 (0.0007)
**SF 36 RP**			
T0	53.4 (46.1)	49.0 (44.4)	Time effect: 5.73 (0.006)
T1	56.0 (40.0)	64.6 (33.2)	Group effect: 0.20 (0.656)
T2	56.9 (38.0)	66.4 (31.5)	Interaction group × time: 2.78 (0.071)
**SF 36 BP**			
T0	75.5 (22.4)	70.2 (26.2)	Time effect: 0.96 (0.389)
T1	71.9 (20.3)	77.2 (20.7)	Group effect: 0.30 (0.585)
T2	70.8 (21.9)	80.0 (19.5)	Interaction group × time: 7.93 (0.001)
**SF 36 GH**			
T0	63.9 (15.9)	61.7 (17.1)	Time effect: 2.51 (0.091)
T1	60.5 (15.0)	72.3 (11.5)	Group effect: 4.91 (0.031)
T2	59.3 (16.8)	73.8 (11.6)	Interaction group × time: 10.87 (0.0001)
**SF 36 VT**			
T0	64.3 (20.3)	61.0 (18.2)	Time effect: 4.16 (0.021)
T1	61.4 (19.7)	72.8 (11.4)	Group effect: 3.26 (0.076)
T2	59.9 (20.8)	75.5 (12.0)	Interaction group × time: 13.89 (<0.0001)
**SF 36 SF**			
T0	69.6 (17.0)	61.8 (14.4)	Time effect: 1.46 (0.242)
T1	61.0 (13.2)	76.3 (12.2)	Group effect: 6.79 (0.012)
T2	59.3 (14.1)	77.4 (10.6)	Interaction group × time: 33.25 (<0.0001)
**SF 36 RE**			
T0	66.4 (33.4)	67.5 (29.1)	Time effect: 0.58 (0.562)
T1	59.6 (24.1)	79.1 (22.1)	Group effect: 4.09 (0.048)
T2	59.6 (24.7)	79.4 (20.6)	Interaction group × time: 8.53 (0.0006)
**SF 36 MH**			
T0	69.8 (20.5)	65.6 (17.8)	Time effect: 1.31 (0.279)
T1	63.9 (21.4)	75.5 (16.1)	Group effect: 2.21 (0.143)
T2	63.6 (21.5)	77.7 (16.0)	Interaction group × time: 13.51 (<0.0001)
**IL-6 (pg/mL)**			
T0	6.3 (1.8)	6.0 (2.1)	Time effect: 0.43 (0.652)
T1	6.0 (1.6)	6.2 (2.3)	Group effect: 0.37 (0.545)
T2	6.2 (1.9)	5.5 (2.4)	Interaction group × time: 0.43 (0.652)
**TNF α (pg/mL)**			
T0	5.4 (3.1)	4.5 (3.0)	Time effect: 2.82 (0.068)
T1	3.2 (2.4)	4.6 (3.0)	Group effect: 0.00 (0.953)
T2	3.4 (2.1)	3.2 (3.1)	Interaction group × time: 1.72 (0.189)
**ß CAROTENE (nM)**			
T0	624.3 (361.3)	871.8 (495.2)	Time effect: 4.23 (0.020)
T1	721.8 (387.5)	931 (598.2)	Group effect: 3.45 (0.069)
T2	869.6 (708.0)	1136.4 (1033.9)	Interaction group × time: 0.12 (0.891)
**LYCOPENE (nM)**			
T0	1201.4 (593.8)	1411.7 (678.1)	Time effect: 4.76 (0.0125)
T1	1105.4 (531.6)	1437.5 (715.6)	Group effect: 0.357 (0.86)
T2	1693.0 (1215.9)	1644.0 (922.2)	Interaction group × time: 1.41 (0.254)
**VITAMIN A (nM)**			
T0	1984.2 (529.0)	2081.6 (550.4)	Time effect: 1.32 (0.275)
T1	1919 (804.7)	1947.7 (518.5)	Group effect: 0.00 (0.951)
T2	2047.7 (549.3)	1948.1 (531.8)	Interaction group × time: 1.59 (0.213)
**γ TOCOPHEROL (nM)**			
T0	2088.8 (1459.0)	2496.3 (1438.4)	Time effect: 2.55 (0.087)
T1	2119.5 (1147.8)	2479.0 (1474.2)	Group effect: 2.63 (0.111)
T2	1590.4 (702.0)	2287.2 (1401.3)	Interaction group × time: 0.57 (0.568)
**α TOCOPHEROL (nM)**			
T0	34161.1 (6834.9)	34254.3 (6451.9)	Time effect: 0.59 (0.558)
T1	34071.5 (6230.5)	34067.2 (6796.5)	Group effect: 0.00 (0.970)
T2	33383.1 (7854.5)	33480.7 (7455.3)	Interaction group × time: 0.00 (0.996)
**REACTIVE C PROTEIN (mg/dL)**			
T0	0.3 (0.5)	0.2 (0.2)	Time effect: 1.83 (0.170)
T1	0.5 (0.9)	0.3 (0.6)	Group effect: 0.77 (0.384)
T2	0.3 (0.5)	0.3 (0.4)	Interaction group × time: 0.55 (0.582)

IL-6: interleukin 6; TNF: tumor necrosis factor. Other abbreviations are as in Table 2.

## Data Availability

De-identified individual data might be made available following publication by reasonable request to the corresponding author.

## Supplementary Materials

The following are available online at https://www.mdpi.com/article/10.3390/cancers13081752/s1: Plasma measurements, Table S1: Pearson’s correlation coefficient between psychometric measures and redox status.

## References

[1] Early Breast Cancer Trialists’ Collaborative Group (2011). Effect of radiotherapy after breast-conserving surgery on 10-year recurrence and 15-year breast cancer death: Meta-analysis of individual patient data for 10,801 women in 17 randomised trials. Lancet.

[2] Hall S., Rudrawar S., Zunk M., Bernaitis N., Arora D., McDermott C.M., Anoopkumar-Dukie S. (2016). Protection against Radiotherapy-Induced Toxicity. Antioxidants.

[3] Allemani C., Weir H.K., Carreira H., Harewood R., Spika D., Wang X.-S., Bannon F., Ahn J.V., Johnson C.J., Bonaventure A. (2015). Global surveillance of cancer survival 1995–2009: Analysis of individual data for 25,676,887 patients from 279 population-based registries in 67 countries (CONCORD-2). Lancet.

[4] Terrazzino S., La Mattina P., Masini L., Caltavuturo T., Gambaro G., Canonico P.L., Genazzani A.A., Krengli M. (2012). Common variants of eNOS and XRCC1 genes may predict acute skin toxicity in breast cancer patients receiving radiotherapy after breast conserving surgery. Radiother. Oncol..

[5] Terrazzino S., Cargnin S., DeAntonio L., Pisani C., Masini L., Canonico P.L., Genazzani A.A., Krengli M. (2019). Impact of ATM rs1801516 on late skin reactions of radiotherapy for breast cancer: Evidences from a cohort study and a trial sequential meta-analysis. PLoS ONE.

[6] Liu C., Lin Q., Yun Z. (2015). Cellular and Molecular Mechanisms Underlying Oxygen-Dependent Radiosensitivity. Radiat. Res..

[7] Kim J.H., Jenrow K.A., Brown S.L. (2014). Mechanisms of radiation-induced normal tissue toxicity and implications for future clinical trials. Radiat. Oncol. J..

[8] Mavragani I.V., Laskaratou D.A., Frey B., Candéias S.M., Gaipl U.S., Lumniczky K., Georgakilas A.G. (2016). Key mechanisms involved in ionizing radiation-induced systemic effects. A current review. Toxicol. Res..

[9] Xiao C., Miller A.H., Felger J., Mister D., Liu T., Torres M.A. (2017). Depressive symptoms and inflammation are independent risk factors of fatigue in breast cancer survivors. Psychol. Med..

[10] Bouchard L.C., Antoni M.H., Blomberg B.B., Stagl J.M., Gudenkauf L.M., Jutagir D.R., Diaz A., Lechner S., Glück S., Derhagopian R.P. (2016). Postsurgical Depressive Symptoms and Proinflammatory Cytokine Elevations in Women Undergoing Primary Treatment for Breast Cancer. Psychosom. Med..

[11] Pitman A., Suleman S., Hyde N., Hodgkiss A. (2018). Depression and anxiety in patients with cancer. BMJ.

[12] Mitchell A.J., Chan M., Bhatti H., Halton M., Grassi L., Johansen C., Meader N. (2011). Prevalence of depression, anxiety, and adjustment disorder in oncological, haematological, and palliative-care settings: A meta-analysis of 94 interview-based studies. Lancet Oncol..

[13] Boehm K., Cramer H., Staroszynski T., Ostermann T. (2014). Arts Therapies for Anxiety, Depression, and Quality of Life in Breast Cancer Patients: A Systematic Review and Meta-Analysis. Evid. Based Complement. Altern. Med..

[14] Archer S., Buxton S., Sheffield D. (2015). The effect of creative psychological interventions on psychological outcomes for adult cancer patients: A systematic review of randomised controlled trials. Psychooncology.

[15] Greenlee H., DuPont-Reyes M.J., Rn L.G.B., Carlson L.E., Cohen M.R., Deng G., Johnson J.A., Mumber M., Seely D., Zick S.M. (2017). Clinical practice guidelines on the evidence-based use of integrative therapies during and after breast cancer treatment. CA A Cancer J. Clin..

[16] Li X.-M., Zhou K.-N., Yan H., Wang D.-L., Zhang Y.-P. (2011). Effects of music therapy on anxiety of patients with breast cancer after radical mastectomy: A randomized clinical trial. J. Adv. Nurs..

[17] Haun M., Mainous R.O., Looney S.W. (2001). Effect of Music on Anxiety of Women Awaiting Breast Biopsy. Behav. Med..

[18] Hanser S.B., Bauer-Wu S., Kubicek L., Healey M., Manola J., Hernandez M., Bunnell C. (2006). Effects of a Music Therapy Intervention on Quality of Life and Distress in Women with Metastatic Breast Cancer. J. Soc. Integr. Oncol..

[19] Cheng P., Xu L., Zhang J., Liu W., Zhu J. (2021). Role of Arts Therapy in Patients with Breast and Gynecological Cancers: A Systematic Review and Meta-Analysis. J. Palliat. Med..

[20] Gramaglia C., Gambaro E., Vecchi C., Licandro D., Raina G., Pisani C., Burgio V., Farruggio S., Rolla R., DeAntonio L. (2019). Outcomes of music therapy interventions in cancer patients—A review of the literature. Crit. Rev. Oncol..

[21] World Medical Association Declaration of Helsinki (2013). Ethical Principles for Medical Research Involving Human Subjects. JAMA.

[22] Cox J.D., Stetz J., Pajak T.F. (1995). Toxicity criteria of the Radiation Therapy Oncology Group (RTOG) and the European Organization for Research and Treatment of Cancer (EORTC). Int. J. Radiat. Oncol. Biol. Phys..

[23] Robb S.L., Burns D.S., Carpenter J.S. (2011). Reporting guidelines for music-based interventions. J. Health Psychol..

[24] Spielberger C.D. (1989). Inventario per L’ansia di “Stato” e di “Tratto”, Nuova Versione Italiana Dello S.T.A.I.-Forma Y.

[25] Montgomery S.A., Åsberg M. (1979). A New Depression Scale Designed to be Sensitive to Change. Br. J. Psychiatry.

[26] Beck A.T., Steer R.A., Brown G.K. (1996). BDI-II, Beck Depression Inventory.

[27] Friborg O., Hjemdal O., Rosenvinge J.H., Martinussen M. (2003). A new rating scale for adult resilience: What are the central protective resource behind healthy adjustment?. Int. J. Methods Psychiatr. Res..

[28] Apolone G., Mosconi P. (1998). The Italian SF-36 Health Survey: Translation, validation and norming. J. Clin. Epidemiol..

[29] Grossini E., Farruggio S., Pierelli D., Bolzani V., Rossi L., Pollesello P., Monaco C. (2020). Levosimendan Improves Oxidative Balance in Cardiogenic Shock/Low Cardiac Output Patients. J. Clin. Med..

[30] Bulfone T., Quattrin R., Zanotti R., Regattin L., Brusaferro S. (2009). Effectiveness of Music Therapy for Anxiety Reduction in Women With Breast Cancer in Chemotherapy Treatment. Holist. Nurs. Pract..

[31] Bradt J., Dileo C., Magill L., Teague A. (2016). Music interventions for improving psychological and physical outcomes in cancer patients. Cochrane Database Syst. Rev..

[32] Nilsson U. (2008). The Anxiety- and Pain-Reducing Effects of Music Interventions: A Systematic Review. AORN J..

[33] Lyman G.H., Greenlee H., Bohlke K., Bao T., DeMichele A.M., Deng G.E., Fouladbakhsh J.M., Gil B., Hershman D.L., Mansfield S. (2018). Integrative Therapies During and After Breast Cancer Treatment: ASCO Endorsement of the SIO Clinical Practice Guideline. J. Clin. Oncol..

[34] Howard-Anderson J., Ganz P.A., Bower J.E., Stanton A.L. (2012). Quality of Life, Fertility Concerns, and Behavioral Health Outcomes in Younger Breast Cancer Survivors: A Systematic Review. J. Natl. Cancer Inst..

[35] Rossetti A.C., Paladini M.S., Riva M.A., Molteni R. (2020). Oxidation-reduction mechanisms in psychiatric disorders: A novel target for pharmacological intervention. Pharmacol. Ther..

[36] Larouche D., Hanna M., Chang S.-L., Jacob S., Têtu B., Diorio C. (2016). Evaluation of Antioxidant Intakes in Relation to Inflammatory Markers Expression Within the Normal Breast Tissue of Breast Cancer Patients. Integr. Cancer Ther..

[37] Arathi B.P., Raghavendra-Rao Sowmya P., Kuriakose G.C., Shilpa S., Shwetha H.J., Kumar S., Raju M., Baskaran V., Lakshminarayana R. (2018). Fractionation and Characterization of Lycopene-Oxidation Products by LC-MS/MS (ESI)^+^: Elucidation of the Chemopreventive Potency of Oxidized Lycopene in Breast-Cancer Cell Lines. J. Agric. Food Chem..

[38] Rossetti A., Chadha M., Torres B.N., Lee J.K., Hylton D., Loewy J.V., Harrison L.B. (2017). The Impact of Music Therapy on Anxiety in Cancer Patients Undergoing Simulation for Radiation Therapy. Int. J. Radiat. Oncol. Biol. Phys..

