# Supplementary material for: Psychotherapy with Music Intervention Improves Anxiety, Depression and the Redox Status in Breast Cancer Patients Undergoing Radiotherapy: A Randomized Controlled Clinical Trial
Source: Cancers (Basel). 2021 Apr 7;13(8):1752. doi: 10.3390/cancers13081752 (PMC8067630; doi:10.3390/cancers13081752)
Supplement: Supplementary file 1 [file cancers-13-01752-s001.pdf]

Supplementary Materials: Psychotherapy with Music Intervention Improves Anxiety, Depression and the Redox Status in Breast Cancer Patients Undergoing Radiotherapy: A Randomized Controlled Clinical Trial

Patrizia Zeppegno, Marco Krengli, Daniela Ferrante, Marco Bagnati, Vincenzo Burgio,Serena Farruggio, Roberta Rolla, Carla Gramaglia and Elena Grossini

Plasma measurements

Collection of blood samples

For the determination of glutathione (GSH), malondialdehyde (MDA), interleukin 6 (IL-6), tumor necrosis factor α (TNFα), and of the other antioxidants and inflammation variables, 10 mL blood samples were taken from each donor by using BD Vacutainer tubes (sodium heparin as anticoagulant). Each sample was immediately centrifuged by a refrigerated through a centrifuge (Eppendorf, mod. 5702 with rotor A-4-38) for 10 min, at a speed of 3100 g at 4 °C. The plasma obtained was divided into 5 tubes that were stored at –20 °C at the Clinical Biochemistry Laboratory and at the Physiology Laboratory for further processing.

GSH quantification

GSH measurement was performed by using the Glutathione Assay Kit (Cayman Chemical, Ann Arbor, MI, USA), as previously described [1,2]. For the experiments, each plasma sample was deproteinated by adding an equal volume of meta-phosphoric acid solution to the sample that was centrifuged at 2000 g for 2 min. Thereafter, the supernatant was collected and 50 µl/mL of TEAM reagent was added to each sample in order to increase the pH. Fifty µl of the samples was transferred to a 96-well plate where GSH was detected following the manufacturer's instructions through a spectrophotometer (VICTOR™ X Multilabel Plate Reader), at excitation/emission wavelengths of 405–414 nm. Glutathione was expressed as GSH production (µM). The measurements were performed in triplicate.

Thiobarbituric acid reactive substances (TBARS) quantification

TBARS were determined as MDA release. MDA measurement was performed by using the TBARS assay Kit (Cayman Chemical), as previously performed [1,2]. Briefly, 100 µL of each plasma sample was added to 100 µL of sodium dodecyl sulfate (SDS) solution and 2 mL of the Color Reagent, following the manufacturer's instruction. Each sample was boiled for 1 h and then transferred on ice for 10 min in order to stop the reaction. After this time, each sample was centrifuged for 10 min at 1600 g at 4 °C and then, 150 µL was transferred to a 96-well plate where MDA was detected following the manufacturer's instructions through a spectrophotometer (VICTOR™ X Multilabel Plate Reader), at excitation/emission wavelengths of 530–540 nm. Malondialdehyde was expressed as MDA production (µM). The measurements were performed in triplicate.

Human IL-6 ELISA kit

IL-6 measurement was performed by using the Human IL-6 ELISA kit (Invitrogen Carlsbad, California, USA). For the experiments, 50 µL of each plasma sample and 50 µL of Assay Buffer 1X were added to each well with 50 µL of Biotin-Conjugate and incubated at room temperature on microplate shaker for 2 h. After 2 h, each well was washed 4 times with 400 µL of Wash Buffer and then 100 µL of Streptavidin-HRP was added to each well

and incubated for 1 h at room temperature on microplate shaker. After this time, the plate was washed again, and 100 μL of Substrate Solution (tetramethyl-benzidine; TMB) was added to each well and incubated for 10 min at room temperature in the dark. The enzyme reaction was stopped by adding 100 μL Stop Solution (1 M phosphoric acid) into each well and each plate was read immediately. IL-6 was detected following the manufacturer's instructions through a spectrophotometer (VICTOR™ X Multilabel Plate Reader), by using a wavelength of 450 nm. The value of each sample was quantified in respect to IL-6 standard curve and expressed as pg/mL. The measurements were performed in duplicate.

Human TNFα ELISA kit

TNFα measurement was performed by using the Human TNFα ELISA kit (Invitrogen). Briefly, 50 μL of each plasma sample and 50 μL of Sample Diluent were added to each well, with 50 μL of Biotin-Conjugate and incubated at room temperature on microplate shaker for 2 h. After 2 h, each well was washed 4 times with 400 μL of Wash Buffer and then, 100 μL of Streptavidin-HRP was added to each well and incubated for 1 h at room temperature on microplate shaker. After this time, the plate was washed again, and 100 μL of Substrate Solution (TMB) was added to each well and incubated for 10 min at room temperature in the dark. The enzyme reaction was stopped by adding 100 μL of Stop Solution (1 M phosphoric acid) into each well. TNFα was detected following the manufacturer's instructions through a spectrophotometer (VICTOR™ X Multilabel Plate Reader), using a wavelength of 620 nm. The value of each sample was quantified in respect to TNFα standard curve and expressed as pg/mL. The measurements were performed in duplicate.

Antioxidants and inflammation variables

High sensitivity C-Reactive Protein (CRP) was analyzed on ADVIA® 1800 Clinical Chemistry Analyzer (Siemens Healthcare Diagnostics). α and γ tocopherol, lycopene and carotenoids levels were assessed on Agilent Eclipse XDB. Plasma (200 mL) was mixed with 300 μL of water and 500 μL of ice-cold absolute ethanol and immediately vortexed for 10 s. Two millilitres of ice-cold hexane was then added and the mixture was immediately vortexed for 2 min. The mixture was then centrifuged at 3000 g for 1 min and 1 mL of the upper (organic) layer was transferred in a vial, evaporated to dryness under a nitrogen stream and reconstituted with 100 mL of a mixture of acetonitrile/dichloromethane/methanol (67:19:14 by vol.) for HPLC analysis.

This was performed by Agilent 1200 series HPLC with diode array detector and an Agilent Eclipse XDB C18 column (4.6 x 100 mm, 3.5 μm). Twenty microlitres of this mixture was then injected and the antioxidants were eluted with a mobile phase containing acetonitrile/dichloromethane/methanol (67:19:14 by vol.), glacial acetic acid (1 g /L) at a constant flow of 1.8 mL/min. Detection of lipid soluble antioxidants was performed at specific wavelength: retinol (326 nm), γ- and α-tocopherol (292 nm), lycopene and β-carotene (460 nm).

Table S1. Pearson's correlation coefficient between psychometric measures of primary outcomes and redox status.

	GSH (μM)	TBARS (μM)
**STAI I**	−0.05	0.03
**STAI II**	−0.06	0.05
**STAI TOT**	−0.06	0.05
**MADRS**	−0.15	0.01
**BDI**	0.04	−0.01

STAI: State-Trait Anxiety Inventory; MADRS: Montgomery-Asberg Depression Rating Scale; BDI: Beck Depression Inventory; GSH: glutathione; TBARS: Thiobarbituric reactive substances.

References

1. Grossini, E.; Pollesello, P.; Bellofatto, K.; Sigaudo, L.; Farruggio, S.; Origlia, V.; Mombello, C.; Mary, D.A.S.G.; Valente, G.; Vacca. G. Protective effects elicited by levosimendan against liver ischemia/reperfusion injury in anesthetized rats. *Liver Transpl.* **2014**, *20*, 361–375.
2. Grossini, E.; Farruggio, S.; Pierelli, D.; Bolzani, V.; Rossi, L.; Pollesello, P.; Monaco, C. Levosimendan Improves Oxidative Balance in Cardiogenic Shock/Low Cardiac Output Patients. *J. Clin. Med.* **2020**, *9*, 373. doi:10.3390/jcm9020373.
